# Supplementary material for: Effect of Hole Shift on Threshold Characteristics of GaSb-Based Double-Hole Photonic-Crystal Surface-Emitting Lasers
Source: Micromachines (Basel). 2021 Apr 21;12(5):468. doi: 10.3390/mi12050468 (PMC8143113; doi:10.3390/mi12050468)
Supplement: Supplementary file 1 [file micromachines-12-00468-s001.zip › micromachines-1177081-supplementary.pdf]

micromachines

MDPI

Supplementary Materials

Effect of Hole Shift on Threshold Characteristics of GaSb-based Double-Hole Photonic-Crystal Surface-Emitting Lasers

Yu-Hsun Huang [1], Zi-Xian Yang [1], Su-Ling Cheng [1], Chien-Hung Lin [2], Gray Lin [1,*], Kien-Wen Sun [3] and Chien-Ping Lee [1]

[1] Institute of Electronics, National Yang Ming Chiao Tung University, Hsinchu City 30010, Taiwan; a123452001@gmail.com (Y.-H.H.); kkobeandmek@gmail.com (Z.-X.Y.); suling0115@gmail.com (S.-L.C.); cplee@mail.nctu.edu.tw (C.-P.L.)
[2] R&D Division, Phosertek Corporation, Hsinchu City 30010, Taiwan; tony.lin@phosertek.com (C.-H.L.)
[3] Department of Applied Chemistry, National Yang Ming Chiao Tung University, Hsinchu City 30010, Taiwan; kwsun@mail.nctu.edu.tw (K.-W.S.)
* Correspondence: graylin@mail.nctu.edu.tw; Tel.: +886-3-513-1289

1. Plan-view scanning electron microscopy images

Figure S1 shows the plan-view scanning electron microscopy (SEM) images at a magnification of 25,000. The pictures (a) through (p) correspond respectively to Devices A through P listed in Table 1. As we can see from these pictures, the two holes overlapped when the shift in relative positons was less than 120nm (Device F). So the *FF* was lower than the designed value of 10%.

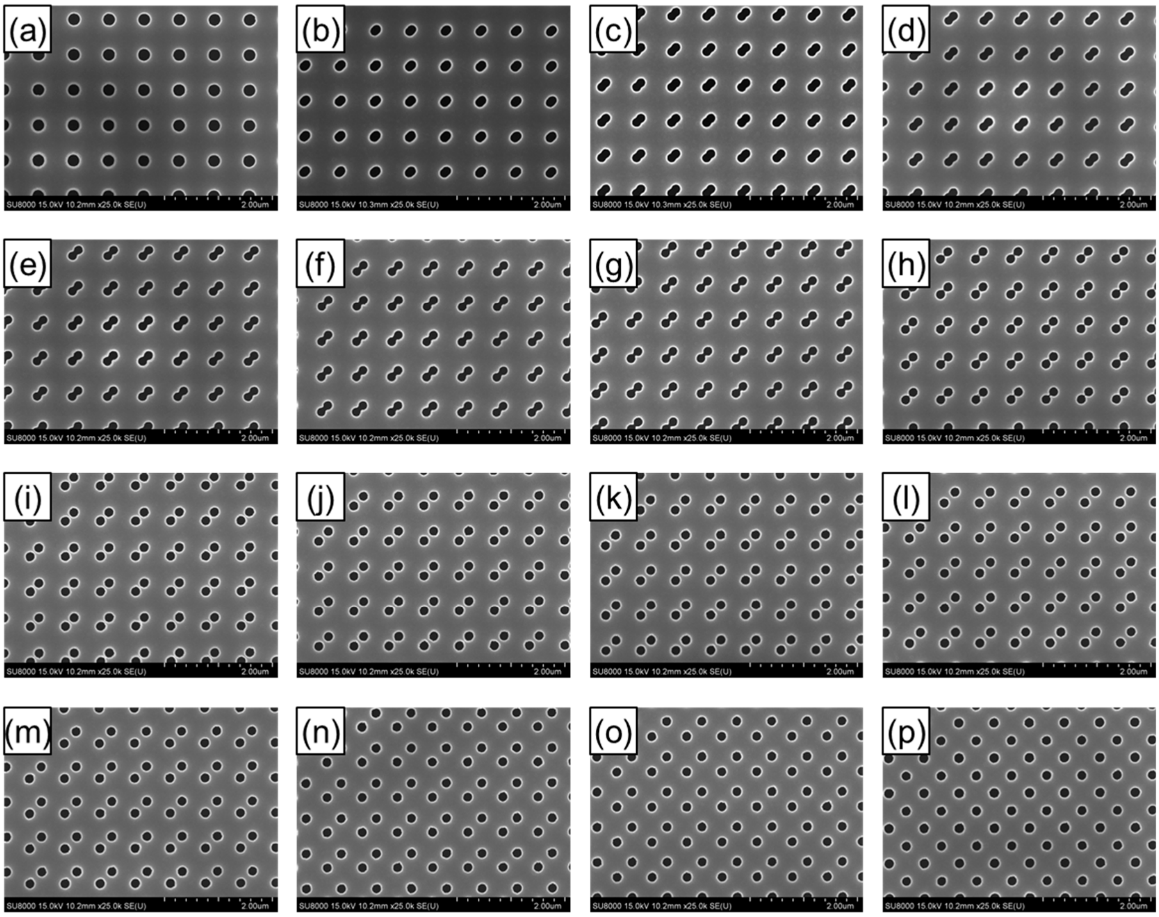

Figure S1. The SEM images for (a) single-hole PC-SEL as well as double-hole PC-SELs with increasing hole-shift distances of (b) 40 (c) 80 (d) 90 (e) 110 (f) 120 (g) 130 (h) 140 (i) 160 (j) 180 (k) 190 (l) 200 (m) 220 (n) 260 (o) 280 and (p) 320 nm.

2. RSoft simulated photonic band-structures

Figure S2 shows the simulated photonic band-structures using the software from RSoft Design Group. The simulation was based on the two-dimensional (2D) plane wave expansion (PWE) method assuming infinite structures. The x-axis was normalized wavenumber in unit of π/a, and the y-axis was normalized frequency in unit of a/λ, where a was the lattice constant. The positive and negative x-axes are along the Γ–M and Γ-X directions, respectively. The effective indices in this simulation are 3.1 and 3.493 for the circular holes and background material, respectively. The filling factors are 10% for both single-hole and double-hole designs.

For the double-hole PC-SELs of Figure S2 (b) to (p), the photonic band-gap initially decreases with increasing hole-shift ratio, then almost vanishes at shift ratio of 1/4, and afterwards increases with increasing shift ratio. The four band-edge modes near the Γ point are designated conventionally, in order of increasing frequency, as ModeA, ModeB, ModeC and ModeD.

The change in band structure is easy to understand using simple Fourier analysis. The magnitudes of the Fourier components depend on the relative positions of the two holes in the unit cell. The second order Fourier component is zero when the shift ratio is 1/4, which the bandgap is zero. In other words, the in-plane forward-backward feed-back diminishes. Because the devices had finite sizes, laser action can still be observed but with a rather high threshold.

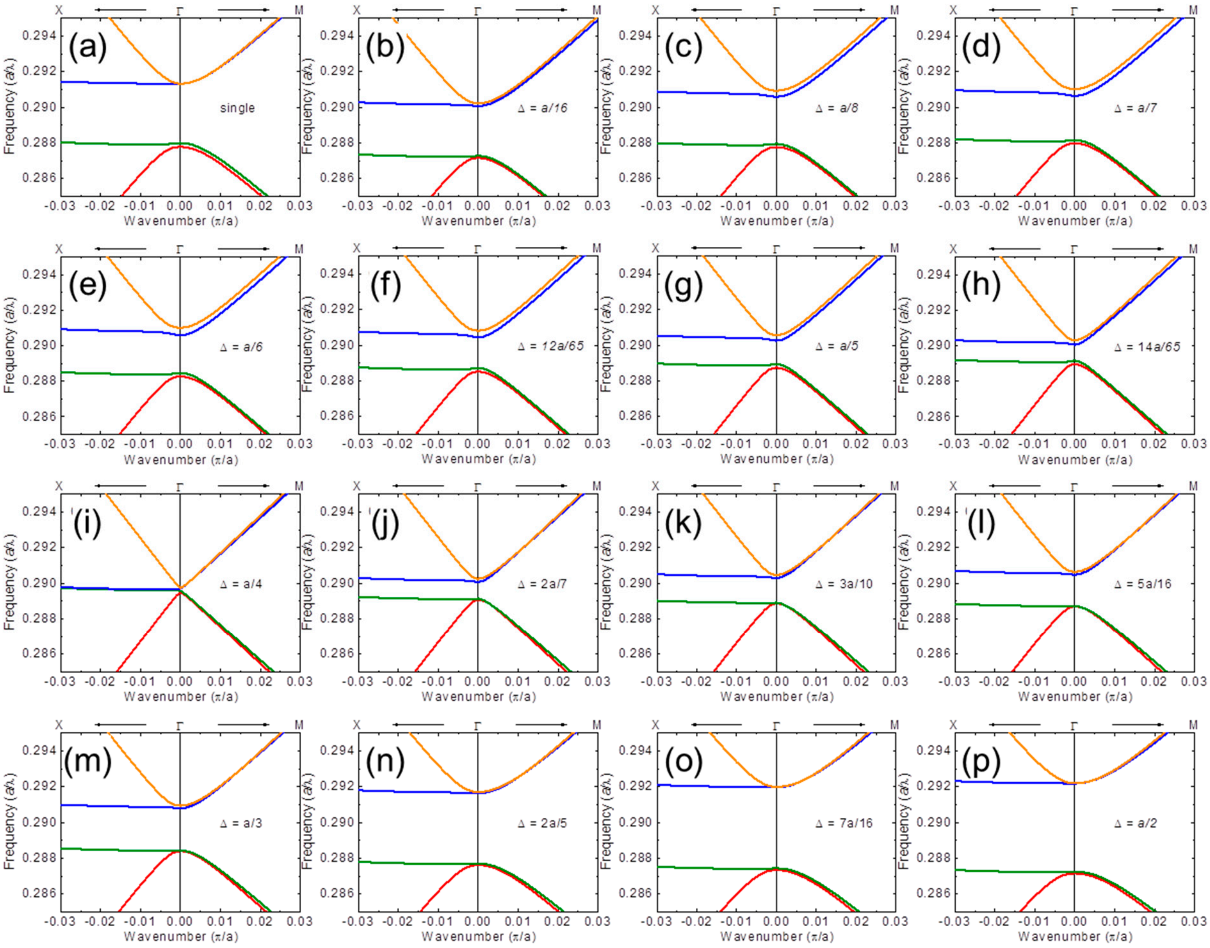

Figure S2. The RSoft simulated band-structures for (a) single-hole design as well as double-hole designs with hole-shift ratio (in terms of lattice period) of (b) 1/16 (c) 1/8 (d) 1/7 (e) 1/6 (f) 12/65 (g) 1/5 (h) 14/65 (i) 1/4 (j) 2/7 (k) 3/10 (l) 5/16 (m) 1/3 (n) 2/5 (o) 7/16 and (p) 1/2.

3. Radiation field distributions, far-field patterns and polarizations

For square-lattice PC with finite cavity size, the radiation field of band-edge modes was obtained by solving eigenvalue equations of three-dimensional (3D) coupled-wave theory (CWT) model [1]. The far-field amplitude was calculated by taking Fourier transform of the radiation field and multiplying by an obliquity factor (cosθ*x* + cosθ*y* - 1) [2]. Figure S3 shows the simulated radiation field as well as far-field intensity distributions of ModeA and ModeD for one single-hole and two double-hole designs (*i.e.* shift ratios of 0, 0.25 and 0.5). For clarity sake, only central region (~ 125 × 125 μm²) out of the 300 × 300 μm² cavity was shown for the radiation field. The arrow lengths and directions indicate the electric field amplitudes and polarizations, respectively.

Given the direction of double-hole shift is along $y = x$ axis, the electric field of ModeA and ModeD in Figure S3 (a) and (b) were both antisymmetric with respect to $y = x$ and $y = -x$ axes. Due to this rotation symmetry, the vertically emitted beam exhibited donut-like pattern. Besides, the double-hole structure with shift ratio of 0.25 introduced totally destructive interference between adjacent two holes, which resulted in additional zero intensity along $y = x$ axis. For double-hole structure with shift ratio of 0.5 in Figure S3 (c), the electric field of ModeA and ModeD were symmetric with respect to $y = -x$ and $y = x$ axis, respectively. However, ModeA exhibit single peak pattern while ModeB exhibited double peak pattern along $y = x$ and $y = -x$ axes. The beam divergence angle of all far-field patterns was around 1°.

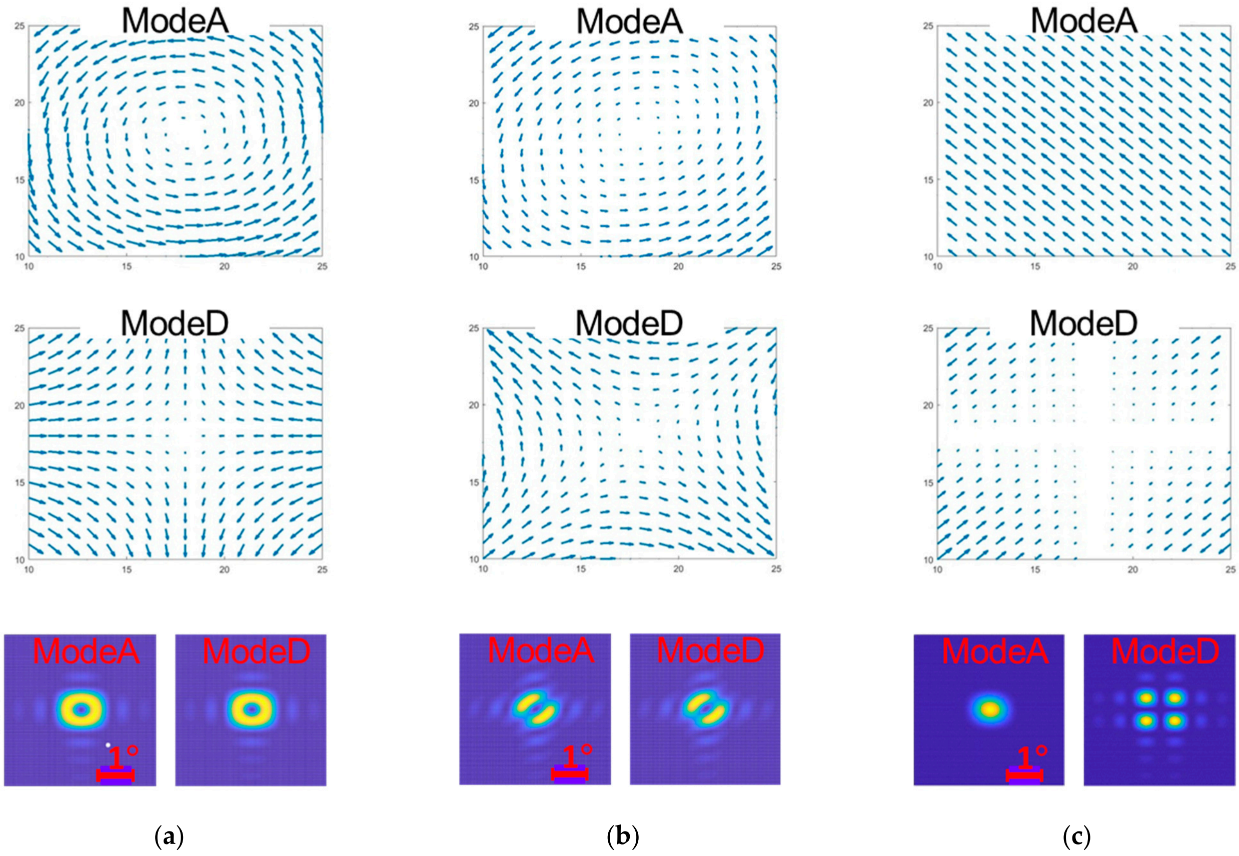

Figure S3. The simulated radiation field as well as far-field intensity distributions of band-edge A and D modes for (a) single-hole design as well as double-hole designs with hole-shift ratio (in terms of lattice period) of (b) 1/4 and (c) 1/2.

References

1. Liang, Y.; Peng, C.; Sakai, K.; Iwahashi, S.; Noda, S. Three-dimensional coupled-wave analysis for square-lattice photonic crystal surface emitting lasers with transverse-electric polarization: finite-size effects. *Opt. Express* **2012**, 20, 15945-15961.
2. Casey, H.C.; Panish, M.B. *Heterostructure Lasers: Part A, Fundamental Principles*; Elsevier, 1978; pp. 71-79.
